# Widespread diffusion changes differentiate Parkinson's disease and progressive supranuclear palsy

**DOI:** 10.1016/j.nicl.2018.09.028

**Published:** 2018-10-04

**Authors:** Aron S. Talai, Jan Sedlacik, Kai Boelmans, Nils D. Forkert

**Affiliations:** a Department of Radiology, Hotchkiss Brain Institute, University of Calgary, Canada; b Department of Diagnostic and Interventional Neuroradiology, University Medical Center Hamburg-Eppendorf, Germany; c Department of Neurology, University Hospital Würzburg, Germany

**Keywords:** Support vector machines, Diffusion-tensor magnetic resonance imaging, Computer-Assisted Image Analysis, Parkinson's disease, Progressive supranuclear palsy, HC, healthy control, PD, Parkinson's disease, PSP-RS, progressive supranuclear palsy – Richardson's syndrome, MNI, Montreal Neurological Institute, MD, mean diffusivity, AD, axial diffusivity, FA, fractional anisotropy, RD, radial diffusivity, ROC, receiver operating characteristic, UPDRS, unified Parkinson's disease rating scale

## Abstract

**Background:**

Parkinson's disease (PD) and progressive supranuclear palsy – Richardson's syndrome (PSP-RS) are often represented by similar clinical symptoms, which may challenge diagnostic accuracy. The objective of this study was to investigate and compare regional cerebral diffusion properties in PD and PSP-RS subjects and evaluate the use of these metrics for an automatic classification framework.

**Material and methods:**

Diffusion-tensor MRI datasets from 52 PD and 21 PSP-RS subjects were employed for this study. Using an atlas-based approach, regional median values of mean diffusivity (MD), fractional anisotropy (FA), radial diffusivity (RD), and axial diffusivity (AD) were measured and employed for feature selection using RELIEFF and subsequent classification using a support vector machine.

**Results:**

According to RELIEFF, the top 17 diffusion values consisting of deep gray matter structures, the brainstem, and frontal cortex were found to be especially informative for an automatic classification. A MANCOVA analysis performed on these diffusion values as dependent variables revealed that PSP-RS and PD subjects differ significantly (*p* < .001). Generally, PSP-RS subjects exhibit reduced FA, and increased MD, RD, and AD values in nearly all brain structures analyzed compared to PD subjects. The leave-one-out cross-validation of the support vector machine classifier revealed that the classifier can differentiate PD and PSP-RS subjects with an accuracy of 87.7%. More precisely, six PD subjects were wrongly classified as PSP-RS and three PSP-RS subjects were wrongly classified as PD.

**Conclusion:**

The results of this study demonstrate that PSP-RS subjects exhibit widespread and more severe diffusion alterations compared to PD patients, which appears valuable for an automatic computer-aided diagnosis approach.

## Highlights

• Brain diffusion was compared in progressive supranuclear palsy-Richardson's syndrome (PSP-RS) and Parkinson's disease (PD).
• Results show widespread diffusion alterations in PSP-RS compared to PD.
• PSP-RS patients show significant reduction of FA and an increase of MD, RD, and AD values in compared to PD.
• RELIEFF feature selection combined with a support vector machine classifier resulted in a differentiation accuracy of 87.7%.

## 1 Introduction

The primary cause of Parkinson's disease (PD) is typically accredited to the accumulation of alpha-synuclein and progressive loss of dopaminergic cells within the substantia nigra (Sharma et al., 2013). Moreover, PD is clinically characterized by a broad range of motor symptoms including bradykinesia, asymmetric rigidity, rest tremor, postural instability, as well as non-motor symptoms such as hyposmia, depression, constipation, and sleep disorder (Singh et al., 2007). In contrast, progressive supranuclear palsy (PSP), an atypical Parkinsonian syndrome, which belongs histo-pathologically to the tauopathies, is distinguished by a vertical supranuclear gaze palsy or slow velocity of vertical saccades, axial rigidity, and repeated unprovoked falls in the early disease course. The clinical diagnosis of PD and PSP is primarily based on medical examinations, response to levodopa, and clinical ratings such as the unified PD rating scale (UPDRS) and others (Hachinski et al., 2006; Hughes et al., 1992; Kalia and Lang, 2015; Se, 1993). However, due to significant overlap of clinical symptoms and inadequate accuracy of bedside tests, differential diagnosis is often challenging, particularly in the early disease course. Within this context, failure rates of up to 24% are reported, even by movement disorders specialists (Hughes et al., 1992). The correct diagnosis, however, is highly critical since disease course, prognosis, and treatment strategies differ between both entities with a significant disadvantage in PSP (Singh et al., 2007). One of the approaches to compensate for these misclassifications are computer-aided diagnosis methods. These recently emerging techniques utilize image and non-image-based information as input features in high level machine learning algorithms for individual level classification of different parkinsonian syndromes and other neurological disorders.

In this context, MRI has gained considerable attention due to its ability to depict abnormalities in the substantia nigra and basal ganglia. Structural T1-weighted MRI sequences can display the macrostructural degeneration profile of different parkinsonian syndromes. In terms of group-wise studies, morphological differences such as white/gray matter volume loss, cortical thickness, and surface area changes have been reported in PD vs. PSP (Duchesne et al., 2009; Price et al., 2004; Worker et al., 2014). In recent studies, volumetric changes in the midbrain, pons area, and cerebral peduncles were reported (Gama et al., 2010; Price et al., 2004; Quattrone et al., 2008). Structural alterations in the cerebellum, thalamus, putamen, pallidum, hippocampus, and brain stem were also shown (Messina et al., 2011). In terms of individual level classification of PD vs. PSP using volumetric features, multiple studies achieved high classification accuracies of >90% (Focke et al., 2011; Salvatore et al., 2014; Sarica et al., 2013; Scherfler et al., 2016). While morphological differences through the use of structural T1-weighted MRI have been extensively employed in group-wise and individual level (classification tasks) studies, other MRI sequences have been less frequently investigated.

Within this context, information extracted from diffusion-tensor MRI (DTI) has been found especially advantageous for examining white matter integrity in various neurological diseases and may identify potential differences at a microstructural level in Parkinsonian syndromes (Hess et al., 2013). Consequently, as microstructural changes are typically expected to precede macrostructural (i.e. volumetric) changes, DTI might indicate brain abnormalities at an earlier stage than structural T1-weighted images. The most relevant quantitative DTI parameters are mean diffusivity (MD), which measures the degree of tissue water diffusivity, fractional anisotropy (FA), an indicator for axonal integrity, radial diffusivity (RD), which is associated with white matter myelin, and axial diffusivity (AD), which provides a metric for axonal degeneration (Song et al., 2002). The typical fingerprint of degenerated neuronal tissue is an increase of MD, RD, and AD but a decrease of FA (Gattellaro et al., 2009; Rizzo et al., 2008).

Nicoletti et al. (2006) reported a significant increase of regional MD values of the putamen, caudate, globus pallidus, thalamus, and the precentral white matter in PSP compared to PD. In two other studies, higher MD values were found in PSP subjects in the superior cerebellar peduncle compared to PD and healthy controls (HC) (Nicoletti et al., 2008; Rizzo et al., 2008). Moreover, the diffusion profile of the superior cerebellar peduncles and corpus callosum have been found to be distinguishing factors in PSP and PD (Agosta et al., 2014; Ito et al., 2008). In another study, higher MD values in globus pallidus and midbrain in PSP compared to PD were reported (Tsukamoto et al., 2012). Furthermore, in line with previous studies, increased MD values in the putamen, globus pallidus, and caudate nucleus in PSP compared to PD were identified (Seppi et al., 2003). Moreover, differences in putamenial longitudinal diffusivity and fractional anisotropy of substantia nigra were reported (Prodoehl et al., 2013). Gattellaro et al. (2009) found that MD values are increased in the substantia nigra, genu of the corpus callosum, and in the superior fasciculus in PD with non-dementia compared to HC. Furthermore, reduced FA values were found in the supplementary motor area, pre-supplementary motor area, and cingulum in PD compared to HC (Karagulle Kendi et al., 2008). Lower FA values in PSP compared to HC in the frontol-orbital area, supplementary motor area, and other areas have also been reported (Erbetta et al., 2009). In addition, a recent study (Rolheiser et al., 2011), found that FA values in the olfactory area are highly beneficial for the differentiation of PD from HC. Furthermore, increased AD and RD values in the substantia nigra, midbrain, and thalamus in PD compared to HC were previously shown (Zhang et al., 2016).

Despite the overwhelming evidence that quantitative DTI parameters have high informative value for differentiating PD from PSP, the aforementioned studies mostly conducted group-wise analyses on a limited number of brain structures while studies utilizing DTI parameters for classification on an individual basis are rather rare (Cherubini et al., 2014; Nicoletti et al., 2006).

Therefore, the present study is focusing on two main objectives. The first goal is to investigate the diffusion properties in a wide range of white and gray matter brain regions in PD and PSP subjects and identify potential differences between the two groups. The second aim is to employ the value of these diffusion maps as input features for an automatic classification of PD and PSP subjects using high-level machine learning techniques.

## 2 Methods

### 2.1 Subjects and MRI sequence specifications

The study cohort used for this work has previously been described (Boelmans et al., 2012). Fifty-two PD and 21 PSP subjects were scanned at the University Medical Center Hamburg-Eppendorf, Germany, using a 3 T Siemens Skyra MR scanner. The clinical diagnosis of PD and PSP was conducted according to the UK Brain Bank criteria (Hughes et al., 1992; Tolosa et al., 2006) and the National Institute of Neurological Disorders and Stroke and Society for PSP (NINDS-SPSP) (Litvan et al., 1996), respectively. The inclusion criteria for the PSP group were probable PSP subjects presenting as classical Richardson's syndrome (PSP-RS) with vertical supranuclear gaze palsy or slow velocity of vertical saccades, axial rigidity, and repeated unprovoked falls within the first three years of the disease. PSP patients with progressive gait freezing, Parkinsonism with tremor or asymmetry or cognitive dysfunction in language or behavioral presentation were excluded. Prior to the study, informed consent was attained from all subjects. The study was approved by the local ethics committee.

Among others, the imaging protocol contained a high-resolution T1-weighted MPRAGE dataset and a DTI dataset. The high-resolution T1-weighted MPRAGE dataset was acquired using TR = 1900 ms, TE = 2.46 ms, flip angle = 9°, TI = 900 ms, image in-plane resolution of 0.94mm^2^, and 0.94 mm slice thickness. The DTI sequence was acquired using a single-shot balanced echo-planar imaging sequence with TR = 4500 ms, TE = 83 ms, and flip angle = 90°. The DTI sequence consists of 27 contiguous transverse slices with a slice thickness of 5 mm and in-plane resolution of 1.875mm^2^ acquired without diffusion gradients (b = 0 s/mm^2^) and with diffusion gradients (b = 1000s/mm^2^) applied along 20 non-collinear directions, averaged over two acquisitions.

### 2.2 Image processing

**Fig. 1:**
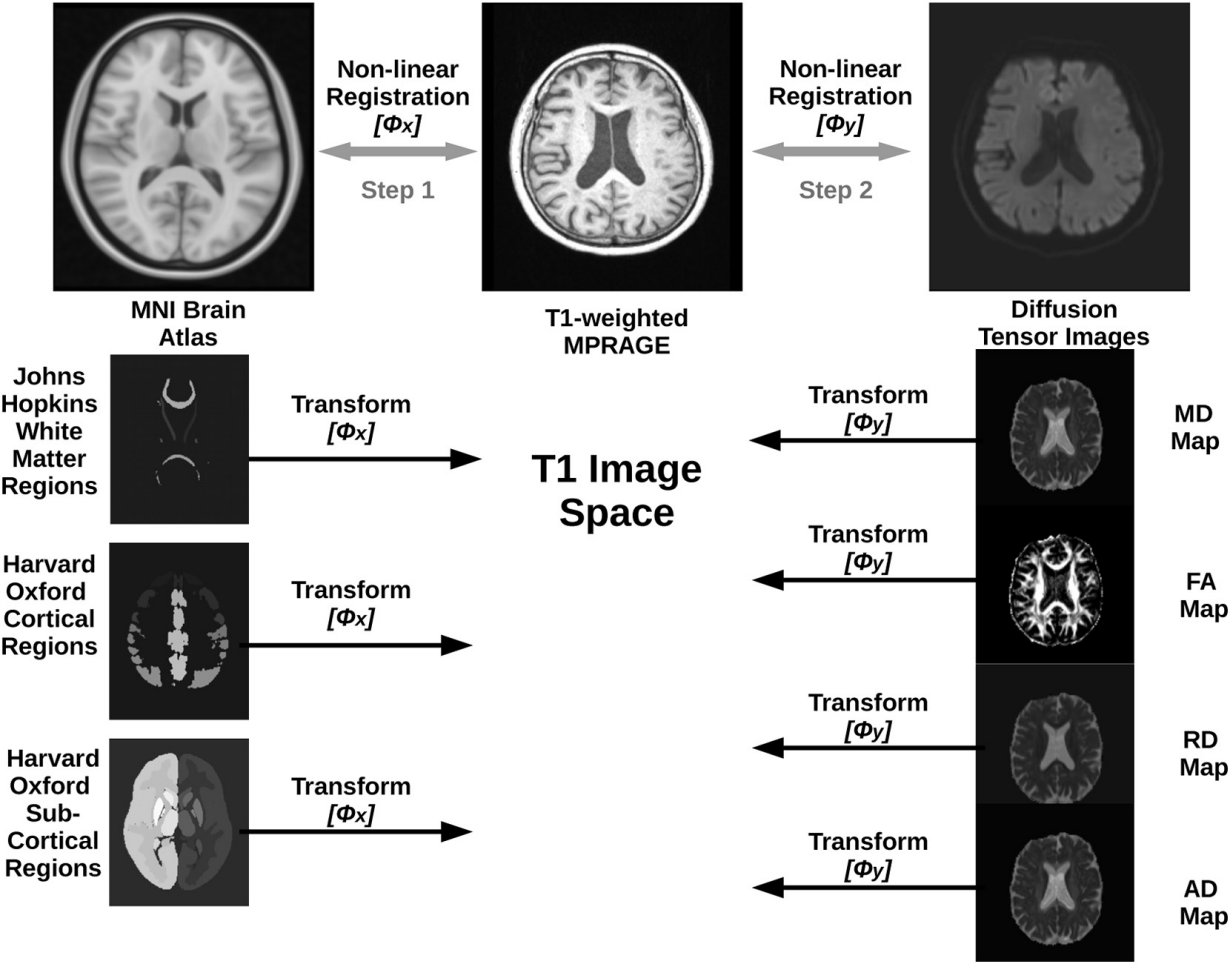
Schematic overview of the image processing phase.

The automatic segmentation of anatomical brain regions was performed by registration of the Montreal Neurological Institute (MNI 152) brain atlas to each T1-weighted MPRAGE image. Afterwards, the DTI sequence was also registered to the MPRAGE image. The image processing pipeline for extraction of the regional MD, FA, RD, and AD values, described in the following, is illustrated in Fig. 1.

In detail, the MNI atlas was registered to the patient-specific MPRAGE images using a rigid followed by an affine transformation. The resulting affine transformation was then used as an initialization for non-linear registration, which was performed using a free-form deformation (Rueckert et al., 1999). The Harvard-Oxford cortical, Harvard-Oxford subcortical, and the Johns Hopkins University white matter tractography atlas brain regions were transformed into the T1 space using the transformation obtained from the MNI to T1 registration. Similarly, each patient-specific DTI dataset, after eddy current distortion correction, was registered to the corresponding MPRAGE dataset using another non-linear registration. This non-linear registration was used to compensate for the inherent DTI-B0 distortion effects. More precisely, the b = 0 s/mm^2^ DTI image was used as the reference for this due to improved anatomical details and higher similarity to the T1-weighted MPRAGE dataset. This registration also consisted of a rigid followed by an affine transformation used for initialization of the non-linear registration, which was performed using a symmetric diffeomorphic image registration method (Avants et al., 2008).

The DTI preprocess tool was used for DTI processing to generate the diffusion parameter maps (MD, FA, RD, AD) (Jenkinson, 2003). The diffusion parameter maps were transformed to the MPRAGE dataset using the corresponding non-linear transformation. The transformed brain atlas regions from the first registration step were then used to determine median diffusion parameters so that 516 DTI features are available for each patient. Median instead of average values were used to account for potential non-normal DTI parameter value distribution and partial volume effects at the border of brain structures.

### 2.3 Statistical analysis, feature selection and classification

A multivariate analysis of covariance (MANCOVA) was used for group comparison using a subset of the median MD, FA, RD, and AD values as dependent variables, age as a co-variate, and the class (PD vs. PSP) as the fixed factor. The subset of values investigated here (see below for details) were the top performing features obtained by the RELIEFF feature selection method (Kononenko et al., 1997). Additionally, a receiver operating characteristic (ROC) analysis was performed for statistical evaluation of each diffusion parameter in the investigated subset. IBM SPSS Statistics (v22.0, IBM, Armonk, NY) was used for all conventional statistical analyses. A *p*-value <.05 (Bonferroni corrected) was considered significant.

Apart from conventional statistical analyses, an individual level differentiation between PD and PSP was also performed using the entire feature set. The classification procedure used in this work starts with a feature ranking routine. Feature selection is often employed to remove redundant and non-informative features from the feature space where they often decrease classification accuracy (Kwak and Choi, 2002). In this work, the RELIEFF feature selection algorithm was used due to its ability to detect conditional dependencies and overall noise robustness.

After feature ranking using the aforementioned method, a linear kernel support vector machine classifier with the default parameter value C = 1, which controls the trade-off between misclassification and error-minimization, was trained based on the highest ranked features. The linear kernel and the default parameter value C = 1 were selected to reduce the risk of overfitting. The support vector machine was chosen due to its consistent strong performance in a wide range of classification tasks in the past (Meyer et al., 2003).

After training, the leave-one-out cross validation routine was employed for classifier performance evaluation. Furthermore, to prevent double dipping, a nested cross validation was employed, meaning that the leave-one-out cross validation also included the feature ranking as described above so that the optimal features used for the actual classification can vary in each iteration of the leave-one-out cross validation. The optimal number of highest ranked features used for training and testing of the classifier was systematically optimized by iteratively removing the lowest ranked feature from the training and testing.

To investigate the benefit of a full diffusion-tensor MR imaging sequence compared to a more simple and faster diffusion-weighted MRI sequence acquired with just three different orthogonal directions with one diffusion weighting, which can only generate MD parameter maps but no FA, RD, and AD parameter maps, the same RELIEFF feature selection, support vector machine classifier, and leave-one-out cross validation procedure described above was also performed using only the MD values.

## 3 Results

The group-wise characteristics of the 73 subjects included in this study together with the corresponding statistics are shown in Table 1. Overall, the two groups differed significantly in disease duration, UPDRS (ON condition), and mini mental state examination (MMSE) test.

**Table 1:** Demographic and clinical characteristics of study participants.

	Parkinson's disease	Progressive supranuclear palsy – Richardson's syndrome	*p*-value
No.	52	21	
Gender, F/M	17/37	11/10	*p* = .093
Age at examination, y, mean ± SD (range)	65.5 ± 8.6 (40–77)	71.1 ± 5.5 (59–79)	*p* = .104
Disease duration, y, mean ± SD (range)	12.7 ± 6.8 (0.5–30.2)	5.9 ± 3.3 (1.2–12.6)	*p* = .001
Hoehn&Yahr, mean ± SD (range)	2.5 ± 0.8 (1–4)	2.5 ± 0.8 (1–4)	*p* = .823
UPDRS motor score (OFF condition), mean ± SD (range)	36.8 ± 13.1 (14–63)	32.7 ± 11.7 (9–52)	*p* = .658
UPDRS motor score (ON condition), mean ± SD (range)	19.9 ± 10.2 (5–52)	28.7 ± 10.6 (6–48)	*p* = .003
MMSE, mean ± SD (range)	28.1 ± 1.4 (23−30)	25.1 ± 2.7 (19–29)	*p* = .032

### 3.1 Statistical analysis of regional diffusion values

The MANCOVA analysis using the top performing median diffusion values as dependent variables, age as a covariate, and the class as a fixed factor revealed a statistical significant difference between PSP-RS and PD subjects (*p* < .001). Table 2 shows the statistical analysis of the top 17 brain regions, which were found to lead to the best classification results (see below). Generally, PSP-RS subjects exhibited reduced FA, and increased MD, RD, and AD values in nearly all brain structures analyzed compared to PD subjects (full data for all 516 features is not shown). The 17 brain regions shown in this table, including the corresponding *p*-values and ROC-AUC values, were determined using all datasets, while the set and rank of features used for classification may vary for each result of the training procedure of the cross validation. Overall, the 17 best features selected for classification also show highly discriminative statistical values.

**Table 2:** Results of the statistical evaluation of the regional diffusion values (STD = standard deviation, FA = fractional anisotropy (×10^3^), MD = apparent diffusion coefficient (measured in 10^−6^ mm^2^/s), RD = radial diffusivity (measured in 10^−6^ mm^2^/s), and AD = axial diffusivity (measured in 10^−6^ mm^2^/s)) of the top 17 ranked features used for classification. Brain regions are sorted according to the feature ranking with decreasing importance.

Rank	Brain Region	Age-adjusted mean (±STD) in PD	Age-adjusted mean (±STD) in PSP-RS	ROC AUC	95% Confidence Limits		p-value
1	AD Right Pallidum	1196.3 ± 10.9	1350.0 ± 17.5	0.923	0.856	0.991	<0.001
2	MD BrainStem	906.0 ± 9.2	1013.4 ± 14.9	0.882	0.794	0.970	<0.001
3	MD Right Pallidum	834.0 ± 8.2	913.6 ± 13.2	0.830	0.714	0.946	<0.001
4	RD BrainStem	717.8 ± 9.0	820.5 ± 14.6	0.850	0.747	0.952	<0.001
5	AD Right Putamen	981.6 ± 15.0	1073.5 ± 24.2	0.799	0.681	0.918	0.002
6	AD Left Putamen	1261.8 ± 13.0	1363.0 ± 20.9	0.840	0.748	0.931	<0.001
7	AD BrainStem	1367.5 ± 9.1	1460.3 ± 14.7	0.883	0.809	0.957	<0.001
8	RD Right Pallidum	683.8 ± 9.4	729.9 ± 15.2	0.736	0.587	0.886	0.14
9	AD Right Thalamus	1219.4 ± 14.0	1379.2 ± 22.6	0.857	0.758	0.956	<0.001
10	RD Right Superior Frontal Gyrus	754.2 ± 11.0	844.6 ± 17.8	0.840	0.745	0.934	<0.001
11	MD Right Thalamus	926.5 ± 13.9	1071.3 ± 22.4	0.822	0.713	0.932	<0.001
12	RD Right Thalamus	799.1 ± 13.5	930.1 ± 21.7	0.799	0.678	0.921	<0.001
13	MD Left Putamen	997.7 ± 13.3	1079.8 ± 21.4	0.814	0.718	0.910	0.002
14	AD Right Frontal Medial Cortex	1186.9 ± 15.8	1291.3 ± 25.5	0.801	0.689	0.914	0.001
15	MD Right Frontal Medial Cortex	1051.4 ± 15.3	1149.4 ± 24.6	0.801	0.688	0.914	0.001
16	MD Right Superior Frontal Gyrus	846.5 ± 10.4	926.6 ± 16.7	0.839	0.745	0.933	<0.001
17	MD Left Superior Frontal Gyrus	881.8 ± 12.1	981.8 ± 19.4	0.841	0.750	0.931	<0.001

### 3.2 PD vs. PSP-RS classification

The leave-one-out cross-validation revealed that the proposed automatic classification method using the diffusion properties as features performs best if the top 17 highest ranked DTI features are used for classification. With this setup, the classification method is capable of differentiating PD from PSP-RS subjects with an overall accuracy of 87.7% (64/73 datasets were correctly classified). More precisely, 6/52 PD subjects were falsely classified as PSP-RS, and 3/21 PSP-RS subjects were falsely classified as PD, which corresponds to a precision of 0.94 for the PD group and 0.75 for the PSP-RS group. The extended classification metrics for all three classification models including true positive rates, Matthews's correlation coefficient (MCC), F-measure, and others are depicted in Table 3.

**Table 3:** Extended classification performance by class (TP = True Positive, FP = False Positive, MCC = Matthews correlation coefficient, ROC = Receiver operating characteristic).

Class	TP Rate	FP Rate	Precision	Recall	F-Measure	MCC	ROC Area
PD	0.885	0.143	0.939	0.885	0.911	0.715	0.913
PSP-RS	0.857	0.115	0.750	0.857	0.800		

Overall, it becomes apparent that the most important brain regions as determined by the RELIEFF algorithm include only the brainstem, deep gray matter structures (putamen, pallidum, and thalamus) as well as areas of the frontal cortex. FA values, although not selected by the RELIEFF algorithm for the classification, were generally reduced in PSP-RS subjects.

Compared to this finding, the highest classification accuracy achieved using only the MD parameters was significantly lower with only 79.5% achieved using the top 85 features as determined by the RELIEFF algorithm. More precisely, 8/52 PD subjects were falsely classified as PSP-RS and 7/21 PSP-RS subjects were falsely classified as PD, which corresponds to a precision of 0.86 for the PD group and 0.64 for the PSP-RS group. The classification metrics for using only MD values as features are shown in Table 4.

**Table 4:** Extended classification performance by class (using only MD values as features) (TP = True Positive, FP = False Positive, MCC = Matthews correlation coefficient, ROC = Receiver operating characteristic).

Class	TP Rate	FP Rate	Precision	Recall	F-Measure	MCC	ROC Area
PD	0.846	0.333	0.863	0.846	0.854	0.506	0.756
PSP-RS	0.667	0.154	0.636	0.667	0.651		

Permutation-based testing with 1000 iterations revealed that both classification results are significant (*p* < .05).

## 4 Discussion

The finding that PSP-RS subjects exhibit reduced FA, and increased MD, RD, and AD values in nearly all brain structures analyzed compared to PD subjects is generally in line with previous studies (Chung et al., 2009; Erbetta et al., 2009; Gattellaro et al., 2009; Karagulle Kendi et al., 2008; Nicoletti et al., 2008, Nicoletti et al., 2006; Rizzo et al., 2008; Rolheiser et al., 2011; Schocke et al., 2002). However, instead of a localized phenomenon as mostly suggested in previous studies, it rather appears to be a general effect as nearly all brain regions analyzed, white as well as gray matter, revealed reduced FA and increased MD, RD, and AD values in PSP-RS compared to PD subjects. These findings point towards more severe microstructural damages of the brain tissue in PSP-RS compared to PD. This is an interesting finding, especially given the aspect that the PD cohort in this study has a significantly longer disease duration but less severe microstructural damages. The differences in microstructural integrity found comparing PD and PSP-RS groups could explain the increased atrophy rates often identified in PSP-RS compared to PD patients. As microstructural changes are typically expected to occur prior to measurable macrostructural changes, DTI parameters might be more viable as early disease biomarkers to differentiate between PD and PSP-RS (Jolly et al., 2013; Zhang et al., 2013).

The 17 brain regions selected and used for classification included the brainstem and deep gray matter structures such as thalamus, putamen and pallidum, all of which are known to be affected by PD and have previously been identified as important brain regions in the volumetric differentiation of PD vs. PSP-RS (Focke et al., 2011; Gama et al., 2010; Messina et al., 2011; Price et al., 2004; Quattrone et al., 2008; Worker et al., 2014). This might further corroborate our proposition that microstructural changes manifest earlier than or are at least correlated with macrostructural changes, promoting the use of diffusion-based sequences over the traditional volumetric T1-weighted images for differential diagnosis of PD vs. PSP-RS. However, this speculation needs to be investigated in more detail in future studies. The other brain regions identified by the feature selection method are part of the frontal cortex, namely the superior frontal gyrus and frontal medial cortex, which could be related to previously reported differences in the prefrontal dopaminergic system between PD and PSP-RS subjects (Narayanan et al., 2013).

Individual level classification of 52 PD and 21 PSP-RS subjects was performed using median regional MD, FA, AD, and RD values obtained from DTI datasets. The leave-one-out cross validation revealed that the proposed support vector machine classifier using these DTI metrics as features can differentiate PD and PSP-RS subjects with an accuracy of 87.7%. The same classification setup using only MD features achieved a significantly lower classification accuracy of 79.5%, which suggests that the additional diffusion metrics that can be calculated from a full DTI sequence have additional informative value compared to using only the MD parameter even though no FA feature was selected by the RELIEFF algorithm when using all diffusion parameters. It is worth noting that RELIEFF does not depend on *p*-values to rank features and follows a different multi-parametric approach. Therefore, features that do not reach statistical significance in conventional statistics might still be ranked highly as they might have a high informative value only in combination with other parameters. For instance, the median radial diffusivity of the right pallidum has a p-value of 0.14 in the group-wise analysis. Nevertheless, it ranked highly according to RELIEFF feature ranking.

It should be noted that this is not the first work to employ DTI measurements for classification of PD and PSP-RS subjects. Haller et al. (2012) presented an approach to classify PD subjects (*n* = 17) and subjects with atypical forms (*n* = 23) of Parkinsonism using a support vector machine classifier and voxel-wise FA values as features. A correct classification between PD subjects versus subjects with atypical forms was achieved in up to 97.5 ± 7.5%. However, it should be noted that the group of 23 subjects with atypical forms of Parkinsonism included only one patient with PSP while the other subjects in this group were, for example, diagnosed with multiple system atrophy, dementia with Lewy bodies, vascular Parkinsonism, and even traumatic brain injury. Thus, the results are not really comparable to those described here. Furthermore, using voxel-wise features for classification always bares the risk of overfitting (Kriegeskorte et al., 2009). Nevertheless, in line with the findings of this study, Haller et al. found decreased FA and increased RD and MD values in the 23 subjects with atypical forms in the bilateral network predominantly in the right frontal white matter compared to PD subjects. Deep gray matter structures were not analyzed in the study by Haller et al. so that no comparison can be made for these brain structures.

The support vector machine is a very powerful machine learning approach and is frequently used for the classification of neurological diseases based on image-based features. Within this context, support vector machines have also been used for the automatic differentiation of PD and PSP patients using morphological features derived from T1-weighted datasets (Focke et al., 2011; Salvatore et al., 2014; Sarica et al., 2013), typically achieving classification accuracies of >90%. The classification accuracy of 87.7% achieved in this work using diffusion measurements, thus, performs comparable to other support vector machine classifiers using volumetric information but might be able to classify patients earlier compared to classifiers relying on macrostructural morphometric information.

It needs to be highlighted that the results of this study and previously presented classification methods are not directly comparable since different databases were used for the development and classifier evaluation. The 73 subjects used in this work, who were recruited prospectively to set up a representative clinical cohort, denote a rather large number of participants compared to most previous studies making it more likely that the results of the proposed classifier are reproducible. It is widely accepted that increasing the number of subjects will reduce the generalization error of the classification model (Figueroa et al., 2012). Classification accuracies obtained from a small number of datasets are often too optimistic and do not necessarily represent the actual classification performance that would be expected in a clinical setting. Thus, the utility of the proposed method needs to be evaluated and validated using a prospective independent study cohort, especially in the context of an early disease marker.

Two major limitations are present in this research. First, the study cohort used in this work, while relatively large compared to similar studies, is still not large enough to fully expand on the generalizability of the proposed model. This limitation is further perpetuated by the lower incidences for PSP-RS compared to PD. Second, an independent validation dataset, preferably acquired in a different imaging center, would be a more rigorous approach of model verification. However, this separate dataset was not available for this present study to further test the proposed model. Moreover, we opted to not separate our current dataset into completely separate training and validation sub groups as the training cohort would not have been sufficiently large enough to train a generalized classifier, potentially resulting in an over-fitted model. Extra precautions such as applying the leave-one-out cross validation and the permutation testing were used to minimize the risk that the results are biased by over-fitting as much as possible. It is worth noting that studies employing separate validation datasets are rather scarce in this context, so that cross validation methods are used most frequently for classifier validation.

In light of the obtained classification accuracy of 87.7%, the proposed method is in the top range of previously reported image-based classification approaches to differentiate PD and PSP subjects using features such as regional brain volumes (Focke et al., 2011), quantitative T2′ values (Boelmans et al., 2012), and susceptibility-weighted MRI parameter (Haller et al., 2013). Despite the favorable outcome of the proposed method, further improvements might be achievable by integrating additional MRI features in the classification method such as susceptibility-weighted imaging, regional brain volumes, or T2 prime MR datasets. Moreover, the usage of DTI datasets with higher spatial resolution as well as the integration of more advanced diffusion imaging sequences, such as diffusion kurtosis imaging or NODDI-DTI, might provide new interesting insights about microstructural differences between the cohorts and improve the differentiation between PD and PSP-RS (Kamagata et al., 2014; Surova et al., 2018). Nevertheless, considering the wide-spread availability of DTI sequences in conventional MRI machines, the presented method portrays a promising new avenue for the diagnosis of PD and PSP-RS. In addition to this, the proposed machine learning model based on regional diffusion metrics could be extended by other atypical Parkinsonian syndromes such as multiple system atrophy, as well as a healthy control group to develop a more comprehensive classification model. However, for this study, we focused solely on the PSP-RS vs. PD classification due to the clinical importance of this differentiation.

In summary, the results of this study demonstrate that regional brain diffusion differences in PD and PSP-RS are present across a wide spectrum of different brain regions, which also enables a high classification accuracy. Moreover, as these micro-structural changes are expected to precede volumetric changes, the DTI sequence might be a more viable tool for the differential diagnosis of PD and PSP-RS compared to structural T1-weighted images.

## Conflict of interest

None.
